# A randomised pilot trial of perioperative propranolol combined with celecoxib versus standard of care in stage III melanoma: The ProCel study protocol

**DOI:** 10.1371/journal.pone.0339476

**Published:** 2026-01-29

**Authors:** Katina J. Selvaraj, Diona L. Damian, J. Guy Lyons, James S. Wilmott, Peter M. Ferguson, Michele McGrady, Iris Bartula, Serigne N. Lo, Richard A. Scolyer, Angela L. Ferguson, Robyn P.M. Saw

**Affiliations:** 1 Faculty of Medicine and Health, The University of Sydney, Sydney, New South Wales, Australia; 2 Royal Prince Alfred Hospital, Camperdown, New South Wales, Australia; 3 Centenary Institute, Camperdown, New South Wales, Australia; 4 Melanoma Institute Australia, The University of Sydney, Sydney, New South Wales, Australia; 5 Charles Perkins Centre, The University of Sydney, Sydney, New South Wales, Australia; 6 NSW Health Pathology, Camperdown, New South Wales, Australia; 7 Mater Hospital, Wollstonecraft, New South Wales, Australia; Fujian Provincial Hospital, CHINA

## Abstract

In cutaneous melanoma patients, the presence of lymph node or in transit metastasis can be associated with poor survival. Adjuvant treatment with BRAF-MEK inhibitors or immunotherapy is costly and can cause side effects, so there remains a need for safe and inexpensive adjuvant treatments. Beta-blockers can control the pro-inflammatory stress response associated with surgery, and their use may attenuate the risk of melanoma progression. COX-2 may promote melanoma progression by increasing prostaglandin production, so COX-2 inhibition may be beneficial. This randomised controlled trial will be conducted at Royal Prince Alfred Hospital and Melanoma Institute Australia, and will include forty participants aged 18–85 years with nodal and/or in transit metastatic melanoma. Participants will be randomly allocated to receive propranolol combined with celecoxib perioperatively, or surgical management only. The primary objective is to determine the effect of propranolol combined with celecoxib on intra-tumoral immune cell populations and tumour proliferative markers, assessed using imaging mass cytometry. The secondary objectives are to evaluate the safety and tolerability of the combination, as well as its effects on: 1) serum inflammatory markers; 2) quality of life; and 3) recurrence/progression rates. The trial is registered in the Australian New Zealand Clinical Trials Registry (ACTRN12624001353583). Protocol version 1 was submitted to PLOS One in February 2025. The trial is supported by Sydney Cancer Partners with funding from Cancer Institute NSW (Grant ID 2021/CBG0002). The sponsors are Sydney Local Health District and Melanoma Institute Australia.

## Introduction

In cutaneous melanoma patients, the presence of lymph node or in transit metastasis is associated with five-year survival rates ranging from 93% in surgically-resected stage IIIA disease to 32% in stage IIID disease [1]. Adjuvant treatment with BRAF-MEK inhibitors or immunotherapy may improve prognosis, but is costly and can cause side effects [2–3]. There remains a need for safe and inexpensive adjuvant treatments to improve survival for patients with stage III melanoma.

### Beta blockade

Surgery is associated with a pro-inflammatory stress response which may promote cancer metastases [4]. Beta-1 and beta-2 adrenoceptors are expressed by several human tumour lines, and there is evidence that epinephrine may play a role in the aetiology of cancer [5]. In melanoma cell lines, epinephrine appears to upregulate tumour progression factors [6]. Beta-blockers can be used to control the physiological sympathetic response that occurs in response to surgery, and their use in melanoma and other cancers appears to attenuate the risk of disease progression [7–8].

### COX-2 inhibition

COX-2 is markedly expressed in poorer prognosis melanomas [9]. COX-2 may promote melanoma progression by increasing prostaglandin (PG) production, stimulating angiogenesis, increasing cell proliferation, and inhibiting apoptosis [10]. PGE_2_, in particular, appears to contribute to cancer progression [11]. Therefore, COX-2 is a potential therapeutic target in the treatment of melanoma. A phase II trial of pembrolizumab and ipilimumab combined with high-dose aspirin (aspirin inhibits COX-2), compared with pembrolizumab and ipilimumab only, resulted in significant survival benefit for patients with advanced melanoma, but was hindered by high rates of adverse events, mostly gastrointestinal [12]. Additionally, responders had differences in cytokine concentrations compared with non-responders, and had increased T regulatory cells, which may indicate improved immune response [12]. These findings suggest that COX inhibition may be of benefit, but safer options are needed.

### Beta blockade combined with COX-2 inhibition

Since both catecholamines and PGs are upregulated perioperatively and can independently promote metastasis, it is plausible that combined beta blockade and COX-2 inhibition may be more effective than either treatment individually [13]. In animal models of post-operative metastasis, the combination has been shown to improve survival [14]. Selective COX-2 inhibitors, which may produce fewer adverse events than aspirin, include etodolac and celecoxib. In a trial of propranolol and etodolac administered perioperatively to patients with colorectal cancer, the combination was well-tolerated and participants who received it had a significant improvement in tumour markers that are associated with progression, including markers of epithelial-to-mesenchymal transition, and a non-significant reduction in recurrence [13].

### Objectives

The primary objective of this study is to determine the effect of perioperative propranolol combined with celecoxib (ProCel regimen) on both intra-tumoral immune cell populations and tumour proliferative markers in patients with nodal and/or in transit metastatic melanoma. The secondary objectives are to evaluate the safety and tolerability of perioperative propranolol combined with celecoxib, as well as its effects on: 1) serum inflammatory markers; 2) quality of life (QoL); and 3) recurrence/progression rates observed clinically and/or through imaging.

## Methods

This is a pilot randomised trial that will be conducted at Royal Prince Alfred Hospital and Melanoma Institute Australia in Sydney, Australia. The trial has been approved by the Sydney Local Health District Ethics Review Committee (2024/ETH00095). Participant recruitment will commence on 01/12/2025. Recruitment is expected to be complete by 31/12/2026, and data collection and results reporting is expected by 31/12/2027. For this pilot study, a sample size of 40 participants (20 receiving the ProCel regimen and 20 receiving standard surgical management) was determined based on previous findings that for comparing human tumours using imaging mass cytometry, 15–20 per arm is sufficient to yield informative effects between arms. The eligibility criteria include adults aged 18–85 years with nodal and/or in transit metastatic melanoma measuring at least 0.5 cm in largest diameter, where surgical resection is expected to render the participant clinically disease free. Patients will be excluded based on the following criteria: allergies or contraindications to the intervention drugs; already taking either intervention drug, calcium channel blockers, or antiplatelet agents; treatment with systemic immunotherapy within four weeks of surgery; internal malignancy; immunosuppression; or being pregnant or lactating. The study will not preclude patients from being offered immunotherapy in the first instance where indicated, and surgical management will not be delayed unnecessarily to facilitate enrolment.

In this pilot, randomised controlled trial, participants will be randomly allocated to receive either the ProCel regimen (Table 1), adapted from a trial of beta blockade and COX-2 inhibition for colorectal cancer [13], or standard surgical management at a 1:1 ratio. New participants will be stratified according to their tumour site (nodal ± in transit metastases, or in transit metastases only), and systemic immunotherapy history (yes or no). A computer-generated randomisation list will be used, with allocation numbers concealed in a password-protected electronic system until a participant is assigned to a group. Participants will be enrolled and assigned by their treating clinician. Participants and treating clinicians will not be blinded. However, investigators analysing tumour tissue and blood samples will be blinded.

**Table 1:** Perioperative propranolol and celecoxib (ProCel) regimen.

	Propranolol	Celecoxib
**Days 1–2**	20 mg twice daily	200mg twice daily
**Days 3–5**	40mg twice daily	200mg twice daily
**Day 6 (day of surgery)**	60mg twice daily	200mg twice daily
**Days 7–13**	40mg twice daily	200mg twice daily
**Days 14–20**	20mg twice daily	200mg twice daily

Participants will self-record heart rate and tablets taken daily. If a participant’s heart rate falls below 60 beats per minute, they continue the current dose without increasing. If a participant experiences dizziness or shortness of breath, they cease propranolol. If a participant develops an unacceptable reaction or intercurrent illness requiring discontinuation of the intervention drugs, they may withdraw from the study. Adherence will be monitored by counting unused tablets. In addition to trial follow-up, participants will receive routine follow-up including clinical assessment, blood tests, and imaging.

**Fig 1:**
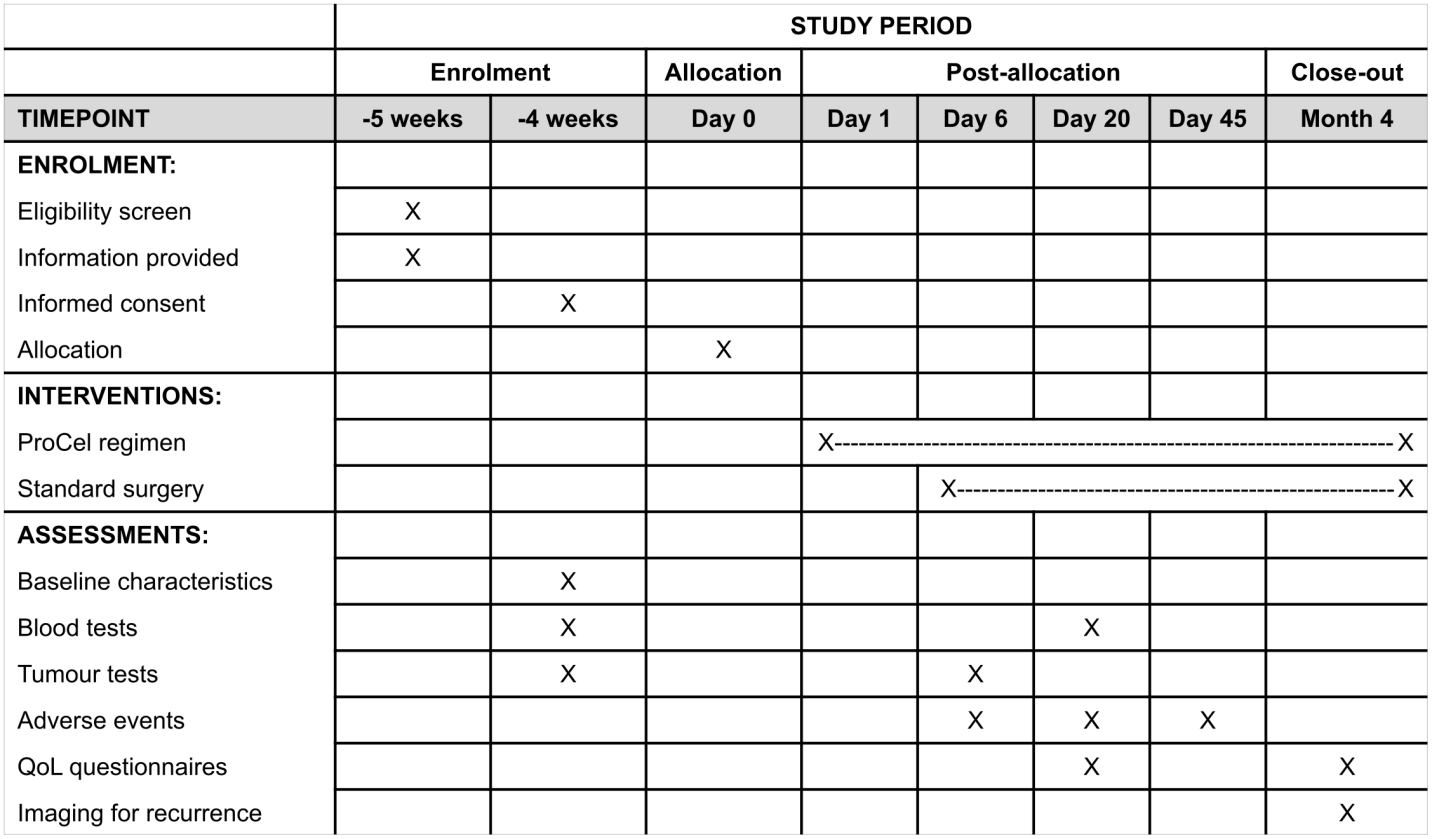
ProCel study SPIRIT schedule.

**Fig 2:**
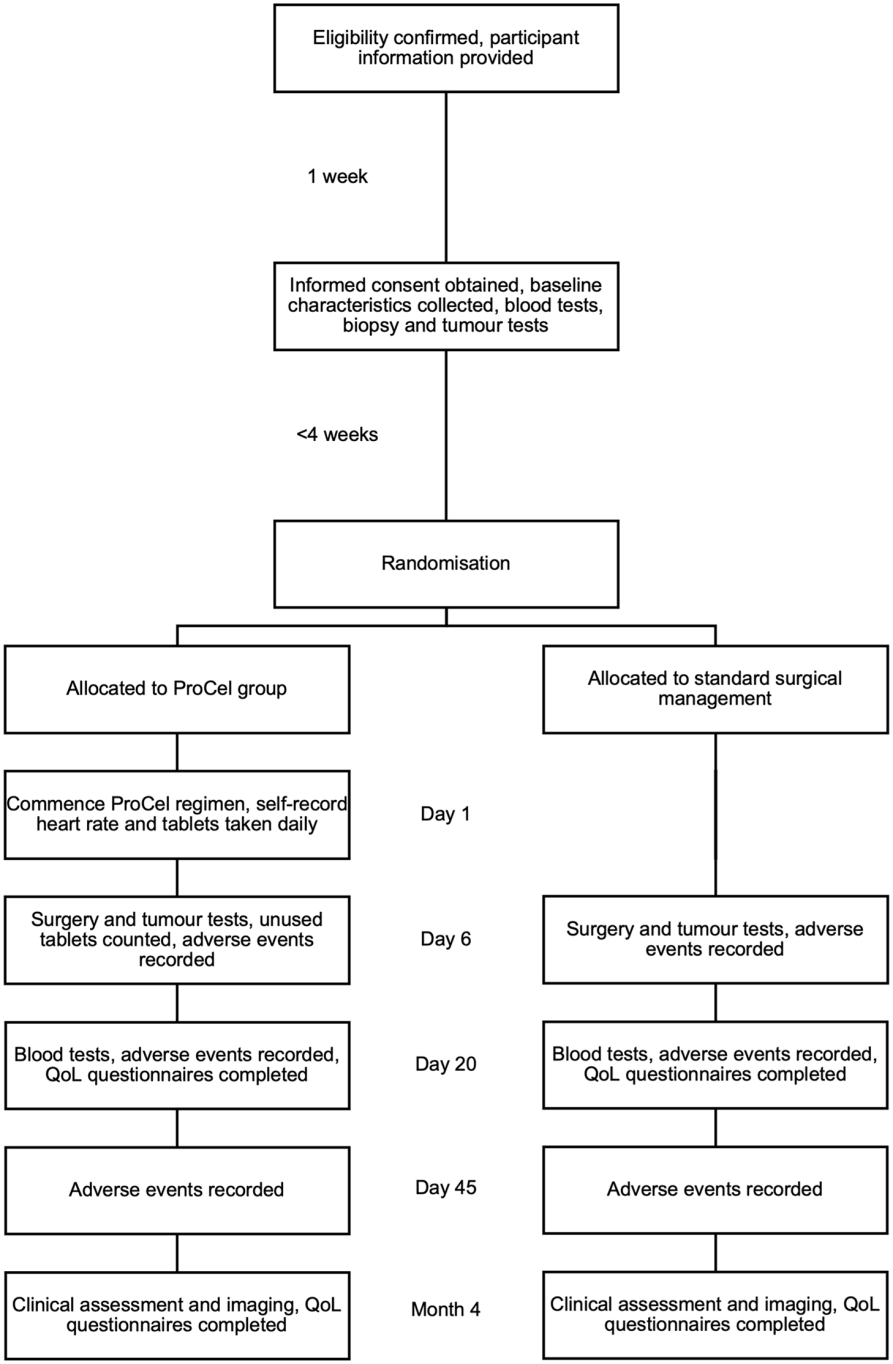
ProCel study participant timeline.

The primary outcome is difference in intra-tumoral immune cell populations and tumour proliferative markers between groups, and changes in these cells when comparing a participant’s biopsied and resected tumour tissue, assessed using imaging mass cytometry. The secondary outcomes are: difference in serum inflammatory markers between groups; difference in QoL scores between groups; rates of adverse events; and rates of recurrence/progression observed clinically and through imaging. The SPIRIT schedule is shown in Fig 1. The participant timeline is shown in Fig 2.

The baseline characteristics to be collected are:

- age (years),
- sex (male/female),
- melanoma stage,
- metastatic site (node/skin),
- largest diameter (mm),
- details of melanoma history,
- previous systemic immunotherapy (yes/no),
- medical history,
- concomitant medications,
- heart rate (bpm),
- blood pressure (mmHg),
- electrocardiogram result (normal/abnormal), and
- QoL questionnaires.

Tumour tests will include histopathology, marker studies using imaging mass cytometry, and mutation profiles (BRAF and NRAS). Studied markers will include B2M-c, CD11c, CD14, CD141, CD163, CD16a-c, CD1c-c, CD20, CD27-c, CD31, CD34, CD38, CD39, CD3e, CD4, CD45, CD45RO, CD68, CD8, CollagenIV, FOXP3, GZMB, HLA-A, HLA-DR, HLA-E, ICOS, IDO1, Ki-67, LAG3, MITF-c, MLANA-c, PD-1, PD-L1, PMEL-c, S100B-c, SMA, SOX10-c, TBET-c, TCF7-c, TIM3-c, Vimentin and VISTA. Blood tests will include a full blood count, electrolytes, urea, creatinine, liver function tests, inflammatory markers, and immune markers. Routine imaging includes positron emission tomography/computed tomography and brain imaging. The QoL questionnaires used are the European Organisation for Research and Treatment of Cancer Quality of Life Questionnaire (EORTC QLQ-C30) [15], the Melanoma Concerns Questionnaire (MCQ-28) [16], the State Optimism Measure [17], and the Perceived Stress Scale [18].

Adverse events will be recorded at each visit, and graded using the National Cancer Institute Common Terminology Criteria for Adverse Events Version 4.0 [19]. Serious adverse events (grade 4 or 5) will be reported to the primary investigator and to the Sydney Local Health District Ethics Review Committee. Participants who withdraw from the trial or discontinue the intervention drug will be asked to complete follow-up to allow collection of outcome data. If a participant discontinues follow-up, their health status will be periodically assessed via phone contact with the participant or their referring doctor.

All data will be analysed according to the intention-to-treat principle. No formal statistical test between groups will be performed as this is a pilot study and not powered enough for detecting significant differences. While minimal loss to follow-up is expected, 15–20 participants per arm is sufficient to yield informative effects between arms, hence the target sample size of 20 participants per arm allows for up to 25% loss to follow-up. Baseline characteristics will be summarised by randomisation arm using descriptive statistics. All primary and secondary outcomes will be analysed by arms. Continuous outcomes will be summarised using mean (standard deviation), and median (interquartile range). Categorical outcomes will be described using their frequency and proportion. Potential effect of ProCel on continuous outcomes (namely intra-tumoral immune cell populations, tumour proliferative markers, serum inflammatory markers, and QoL scores) will be evaluated against standard surgical management by calculating the mean difference between the two groups along with its 95% confidence interval (CI). The difference in rates of adverse events and rates of recurrence/progression between groups will be presented as the relative risk along with its 95% CI.

A data monitoring committee composed of clinicians with appropriate specialty expertise and clinical trials experience will independently monitor patient safety data and provide recommendations if the trial should continue unchanged, undergo modifications or be stopped. The data monitoring committee will report to the investigators and the Sydney Local Health District Ethics Review Committee. If the data monitoring committee recommends stopping the trial, the final decision rests with the investigators. If a member of the data monitoring committee develops a conflict of interest they will resign.

Data will be stored securely in hard copy at Royal Prince Alfred Hospital, and in soft copy in REDCap. It will be accessible only to persons involved in the trial. The investigators will permit independent audits of trial conduct by the Sydney Local Health District Ethics Review Committee, and other relevant regulatory agencies. In addition, the investigators will submit quarterly progress reports to the Sydney Local Health District Ethics Review Committee.

## Ethics and dissemination

Any protocol modifications will be communicated to the Sydney Local Health District Ethics Review Committee, and updated in the ANZCTR.

Participants will be provided with the Participant Information Sheet and Consent Form, and will have at least one week to consider participating. Written informed consent will be obtained from participants, witnessed by a clinician who is not involved in the study. Participants will also be asked to consent to biobanking of their tissue and blood samples in the Melanoma Institute Australia Biospecimen Bank for use in future research.

After participants complete the trial, they will continue routine follow-up with their treating clinician. If participants suffer harm from trial participation, the investigators will assist with arranging appropriate medical treatment. Participants do not give up any legal rights to obtain compensation by participating in the trial.

The investigators will communicate the trial results via publication. The investigators’ access to data and rights to publish are not restricted in any way. When the study is completed and published, deidentified research data will be made fully available as supporting information. There are no restrictions on publicly sharing the deidentified data. Authorship eligibility will be according to the International Committee of Medical Journal Editors’ Recommendations for the Conduct, Reporting, Editing, and Publication of Scholarly Work in Medical Journals [20].

## Supporting information

S1 Checklist ProCel study SPIRIT checklist. (DOC)

S2 Protocol ProCel study protocol. (DOCX)
