# Supplementary material for: A randomised pilot trial of perioperative propranolol combined with celecoxib versus standard of care in stage III melanoma: The ProCel study protocol
Source: PLoS One. 2026 Jan 29;21(1):e0339476. doi: 10.1371/journal.pone.0339476 (PMC12854447; doi:10.1371/journal.pone.0339476)
Supplement: S2 Protocol — (DOCX) [file pone.0339476.s002.docx]

***ProCel Study***

**Perioperative propranolol and celecoxib in stage III melanoma**

**Principal Investigator:**

A/Prof Robyn Saw^1,2,3^

Department of Melanoma and Surgical Oncology, Royal Prince Alfred Hospital; University of Sydney; Melanoma Institute Australia

Ph: +612 9515 5072 Fax: +612 9515 3042 e: robyn.saw@melanoma.org.au

**Protocol Authors:**

Prof Diona Damian^1,2,4^

A/Prof J Guy Lyons^1,5,6^

Dr James Wilmott^1,2,7^

Dr Angela Ferguson^1,6,7^

Dr Peter Ferguson^1,2,8^

A/Prof Michele McGrady^1,9^

Dr Iris Bartula^2^

A/Prof Serigne Lo^1,2^

Prof Richard Scolyer^1,2,7,8^

^1^ Faculty of Medicine and Health, University of Sydney, Sydney

^2^ Melanoma Institute Australia, University of Sydney, North Sydney

^3^ Department of Melanoma and Surgical Oncology, Royal Prince Alfred Hospital, Sydney

^4^ Department of Dermatology, Royal Prince Alfred Hospital, Sydney

^5^ Cancer Services, Royal Prince Alfred Hospital, Sydney

^6^ Centenary Institute, Camperdown, Sydney

^7^ Charles Perkins Centre, The University of Sydney, Sydney

^8^ Tissue Pathology and Diagnostic Oncology, Royal Prince Alfred Hospital, Sydney

^9^ Department of Cardiology, Royal Prince Alfred Hospital, Sydney

**Protocol Version # 1.6 Protocol Date: 17 Apr 2024**

**Ethics Statement:**

The study will be conducted in accordance with the *National Statement on Ethical Conduct in Human Research* (2007), the *CPMP/ICH Note for Guidance on Good Clinical Practice* and consistent with the principles that have their origin in the Declaration of Helsinki. Compliance with these standards provides assurance that the rights, safety and well-being of trial participants are respected. No participants will be identified in any reports, nor will any information identifying any individual subject be included in the data set for analysis.

**CONTENTS**

**Summary 4**

1. **Background and introduction 6**
   1. **Stage III metastatic melanoma 6**
   2. **Surgery cancer press immune competent 7**
   3. **COX-2, catecholamines and carcinogenesis 7**
   4. **β blockers and melanoma 7**
   5. **COX-2 inhibitors and melanoma 8**
   6. **Rationale for performing with study 9**
2. **Hypothesis 10**
3. **Study objectives**
   1. **Primary objectives and endpoint 10**
   2. **Secondary objectives and end points 11**
4. **Study participants 11**
   1. **Inclusion criteria 11**
   2. **Exclusion criteria 12**
5. **Study procedures 13**
   1. **Participants 13**
   2. **Investigational plan 13**
   3. **Participant recruitment and enrolment 16**
   4. **Randomisation procedure 16**
   5. **End of study intervention and withdrawal procedure 16**
6. **Safety 17**
   1. **Serious event reporting 17**
   2. **Adverse events 17**
7. **Confidentiality, storage and archiving of study materials 17**
8. **Trial sponsorship and financing 18**
9. **Audits 19**
10. **Data safety and monitoring board 19**
11. **References 20**

**Appendix 1. Quality of life questionnaires 23**

**Appendix 2. Laoratory tests for Procel 28**

**SUMMARY**

**Study title: Perioperative propranolol and celecoxib in stage III melanoma (ProCel)**

**Objectives**

**Primary Objective**

**To determine the effect of perioperative propranolol and celecoxib on tumoral immune cell infiltrates and proliferation and apoptosis markers in nodal and cutaneous metastatic melanoma compared to control.**

**Secondary Objectives**

To determine the safety and tolerability of the perioperative propranolol and celecoxib (ProCel) regimen

To assess changes in blood markers of inflammation and immunity in ProCel patients versus controls

**Study Design**

Prospective randomised controlled study; patients will be randomised to receive either the ProCel regimen perioperatively (stratified for ±systemic immunotherapy, metastatic site ie node, skin) or no ProCel regimen. For this pilot study placebo tablets will not be used and blinding is not possible for treating clinicians or participants. Investigators analysing tumour tissue and blood samples will be blinded to allocation.

**Planned Sample Size**

**N=40** participants, randomised to receive either the ProCel regimen or standard care.

**Inclusion Criteria**

This investigator-initiated study will be conducted through the Melanoma and Surgical Oncology Clinics at Royal Prince Alfred Hospital and Poche Centre North Sydney. Participants will be attendees of the clinics with histologically confirmed macroscopic nodal or cutaneous melanoma (in transit) metastases awaiting surgery for those metastases. Tumour banked specimens from previous biopsies and surgeries performed on a participant’s melanoma will be accessed from the Melanoma Institute Australia Tumour Bank.

**Study procedure**

Stage III metastatic melanoma in nodes and/or skin (in transit disease)

2 x baseline 2mm^2^ core biopsies of nodes and/or skin (allowing 2 x 1mm^2^ cores from each sample for tissue microarrayanalysis for proliferative, immune and inflammatory markers)

**Baseline**

**bloods, inflammatory**

**and immune markers**

**within 4 weeks of**

**study commencement**

**Randomised 1:1**

Treatment arm: 20 day ProCel regimen for 5d preoperatively, operative day and 14d postoperatively

Control arm

Surgical excision of metastasis as per clinical practice with routine paraffin sections and 1mm^2^ cores x 2 from each sample for tissue microarray analysis for proliferative, immune and inflammatory markers

Bloods for inflammatory and immune markers 2 weeks postoperatively

**Statistical considerations**

**Sample size calculation**: N=40; randomised 1:1 to ProCel or control arms

**Randomised controlled study**

**Duration of the Study** 24 months

# **1. BACKGROUND AND INTRODUCTION**

### **1.1. Stage III metastatic melanoma**

The presence of lymph node metastases or in transit metastases (metastases between the primary melanoma site and local lymph node field) in cutaneous melanoma patients is associated with five-year survival rates ranging from 93% in surgically resected American Joint Committee on Cancer (AJCC 8^th^ edition) stage IIIA disease to 32% for patients with resected stage IIID melanoma^1^. Particularly for stage IIIC and IIID patients the prognosis is dismal. The use of adjuvant drug therapy in the form of BRAF/MEK inhibitors or immunotherapy have improved prognosis, albeit with significant monetary cost and potentially toxic side effects. There is a however, still a need for further improvement in survival in resected stage III melanoma patients, especially using adjuvant agents with low morbidity and low cost.

**1.2. Surgery can suppress immune competence and promote cancer metastasis**

Surgery is life-saving and indispensable, but there have been suggestions that resection of a tumour may increase the risk of metastases for several reasons. It is possible that tumour cells may be shed during physical manipulation of the tumour during surgery^2-5^, or that release of growth factors^6,7^ and pro-angiogenic factors^8-10^ after tissue damage may promote the development of micrometastases. Immune suppression is also present postoperatively, in particular cell mediated immunity with suppression of natural killer cells and cytotoxic T lymphocytes^11,12^ . The perioperative immune suppression appears to correspond to the extent of surgical trauma and tissue damage^13^.

### **1.3. COX-2, catecholamines and carcinogenesis**

### Pro-inflammatory stress responses (especially following surgery) such as production of prostaglandins and catecholamines are linked to promotion of cancer metastasis, by release of angiogenic factors, stimulation of epithelial to mesenchymal transition and suppression of antitumour immunity^14^.

Cyclooxygenase-2 (COX-2) is generally upregulated only in pathological conditions such as inflammation and cancer. The main effects of COX-2 appear to be related to increased prostaglandin production, especially PGE_2,_ which significantly contributes to the induction of inflammation, and tumorigenesis. This has in turn been linked to the simulation of angiogenesis, inhibition of apoptosis, increased cell proliferation and immunosuppression^15-17^ which all assist carcinogenesis. PGE_2_ appears to be upregulated during and after surgery^18^.

**β1**and **β2** adrenoceptors are expressed by several human tumour lines, and catecholamines are potent direct stimulator of migration of some human carcinoma cell types (e.g. colon, breast and ovary) and of secretion of pro-angiogenic factors by these tumours^10,19-21^. Surgical manipulation and accompanying stress responses have been shown to be major suppressors of antimetastatic host cell mediated immunity e.g. cytotoxic activity of natural killer cells decreases following surgery^22^.

Controlling exaggerated inflammatory responses (with COX-2 inhibitors) and physiological (and psychological) sympathetic responses (with **β** blockers) could contribute to prevention of tumour metastatic development^22^. The use of COX-2 inhibitors could also improve postoperative pain and the use of **β** blockers could also assist in decreasing stress and anxiety in patients awaiting surgery.

### **1.4. β blockers and melanoma**

There is a suggestion that norepinephrine, a stress hormone produced after activation of the sympathetic-adrenal-medullary axis might play a role in the aetiology of some types of cancer, melanoma included^23^. It appears to upregulate tumour progression factors in melanoma cell lines^24^ and the use of β blockers in other cancers and in melanoma appears to attenuate the risk of disease progression.^25-27^

**1.5. COX-2 inhibitors and melanoma**

### UV damage of the skin appears to be related to DNA damage and COX-2 mediated PGE_2_ activity^28^. The simulation of angiogenesis, inhibition of apoptosis, increased cell proliferation and immunosuppression linked to COX-2 may promote melanoma initiation and progression^15,16,17^.

The main effects of COX-2 in melanoma appear to be related to PGE_2_ production which appears to contribute to invasion and progression of disease^15^. These are closely linked to the mitogen-activate protein kinase (MAPK) pathway which is a target for BRAF and MEK melanoma drugs^29^. COX-2 expression in melanoma also positively correlates with programmed death-ligand 1 (PD-L1) expression in BRAF mutated melanomas via IL-1 upregulation^30,31^. High PD-L1 expression in many cancers correlates with a bad prognosis, but for melanoma this is controversial.

COX-2 has also been identified as a potential marker for prognosis in melanoma. It has been found to be more markedly expressed in metastatic, poorer prognosis primary melanomas^32-34^ and has been associated with poor prognostic histopathological features like increased Breslow thickness, presence of ulceration and higher mitotic rate^33,35^. COX-2 has an intense expression at the centre of the tumour, with reduced COX-2 at the periphery.

COX-2 may therefore potentially be a therapeutic target in the treatment of melanoma. COX-2 activity can be inhibited by nonsteroidal anti-inflammatory drugs such as naproxen or indomethacin (nonselective inhibition as they also suppresses COX-1 activity) and selective inhibitors like celecoxib (brand name Celebrex)^36^. COX-2 inhibitors have little or no effect on coagulation and are therefore attractive for use in the surgical setting^37^.

### **Table 1** (from Tudor et al^38^) showing the main COX-2 inhibitors tested in melanoma and their effect as possible therapeutic adjuvants.


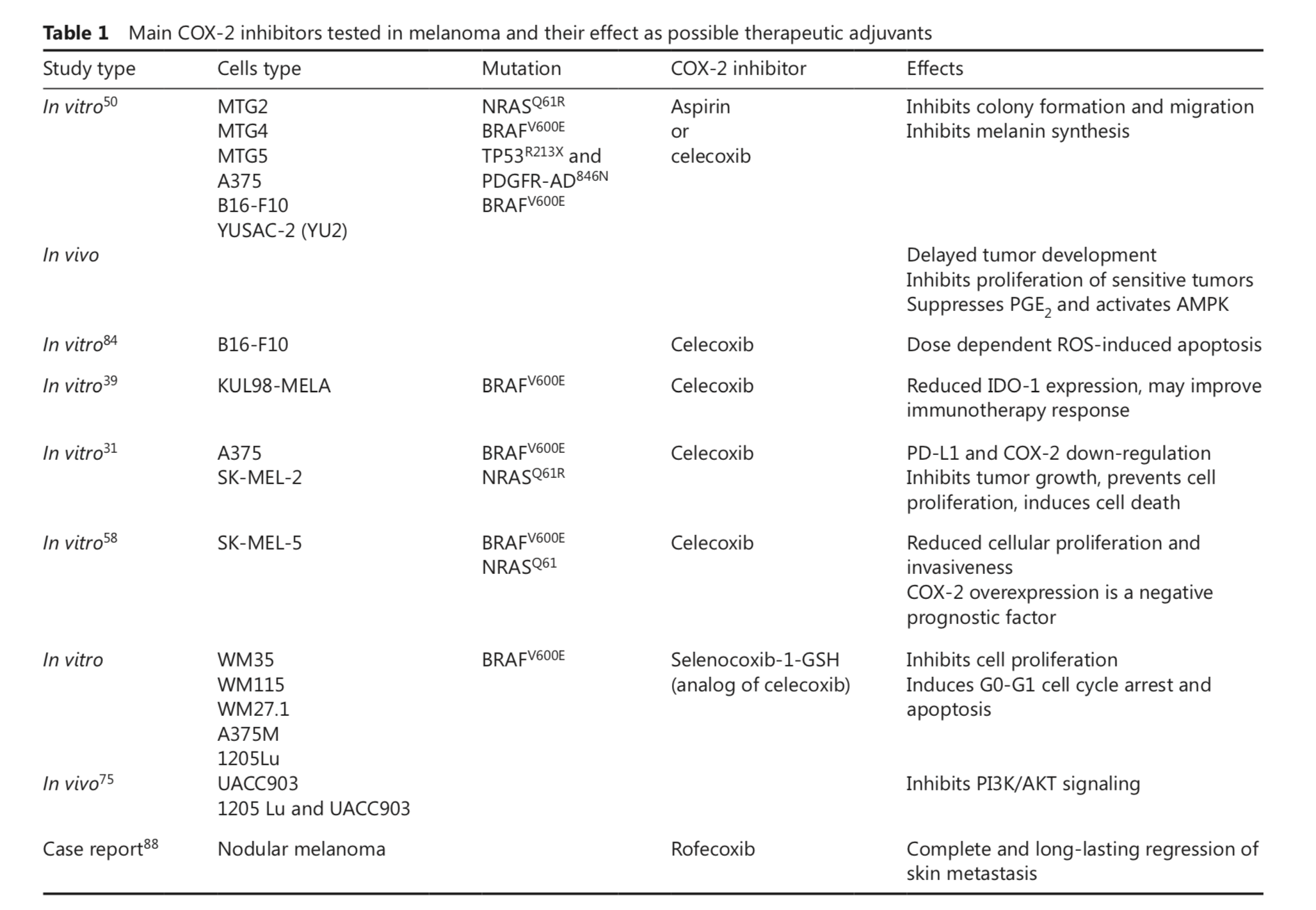


**1.6 Links between psychological stress and immunity**

Chronic psychological stress is associated with neuroendocrine and sympathetic nervous system activation and with suppression of anti-tumour immunity (reviewed by Hong et al)^39^.

### **1.7. Rationale for performing this study**

Despite the availability of systemic immune therapies for metastatic melanoma, such as PD1 inhibitors and ipilimumab, complete response occurs only in a minority of patients. Haldar et al^40^ administered oral propranolol (a β blocker) and the COX-2 inhibitor etodolac for 5 preoperative and 15 postoperative days in patients with colorectal cancer. Compared to patients receiving placebo, patients receiving the propranolol/etodolac combination showed significant changes in tumour markers for epithelial to mesenchymal transition and tumour immune markers. Tumour recurrence rates at 3 years postoperatively were non significantly reduced in the intervention arm (0/11 recurrences with treatment versus 5/17 recurrences with placebo in treatment-compliant patients).

Addition of a brief perioperative regimen of COX2 inhibitor and propranolol (using clinically feasible doses equivalent to those used by Haldar for colorectal cancer) could potentially improve anti-tumour immunity and effect long term influence on recurrence risk. This study will determine whether there are signals for enhanced anti-tumour immunity and reduced proliferation in patients with resected stage III melanoma receiving the ProCel regimen. This data could then be used to inform the design of larger randomised controlled clinical trials.

**2. HYPOTHESIS**

We hypothesise that perioperative administration of the ProCel regimen will alter the tumour immune environment, with reduced immune suppression, and will reduce tumour proliferative markers such as Ki67. We hypothesise that patients receiving the perioperative ProCel regimen will have lower levels of inflammatory markers and elevated markers of immunity in blood postoperatively, and lower circulating tumour DNA 4 months postoperatively, providing a signal that ProCel may reduce recurrence risks and improve outcomes in patients with metastatic melanoma.

# **3. STUDY OBJECTIVES**

### **3.1. Primary Objective/ primary endpoint**

To determine the effect of perioperative propranolol and celecoxib on intra-tumoral and peritumoral immune cell populations and proliferative markers, in patients with melanoma metastatic to nodes and/or in transit disease.

### **3.2. Secondary Objectives/ endpoints**

The secondary objectives are to determine the effect of the ProCel regimen on

3.2.1 Safety and tolerability (adverse events)

3.2.2 Serum inflammatory markers at D14 postoperatively (D20-D27)

3.2.3 Quality of Life, perceived stress and state (modifiable) optimism at D14 and at 4 months postoperatively, and correlation with baseline and postoperative blood and tumour inflammatory and immune markers.

3.2.4 Evidence of disease recurrence/progression at 4 months postoperatively in the 2 arms (based on staging investigations performed as part of routine clinical care. This usually includes PET-CT and brain imaging 3-4 monthly postoperatively and this data will be available for patients in both arms of the study.

# **4.** **STUDY PARTICIPANTS**

This study will be conducted through the Department of Melanoma and Surgical Oncology at Royal Prince Alfred Hospital and Poche Centre, North Sydney. Participants will be attendees of the clinics who require surgical management of their stage III metastatic melanoma.

### **4.1. Inclusion Criteria**

There will be NO EXCEPTIONS to eligibility requirements at the time of randomisation. Questions about eligibility criteria should be addressed PRIOR to enrolment.

Participants must meet ALL of the following inclusion criteria:

4.1.1 Aged 18 years or older, but not older than 85 years

4.1.2 Known metastatic cutaneous melanoma in lymph nodes and/or in transit recurrences in skin, measuring at least 0.5cm in largest diameter, appropriate for surgical management and where surgery is expected to render the patient clinically disease free.

4.1.3 Willingness and ability to provide informed consent and willingness to participate and comply with the study requirements.

### **4.2. Exclusion Criteria**

4.2.1 Unable to follow up for the duration of the study due to frailty, geographical or social reasons

4.2.2 Pregnant or lactating (women of childbearing potential must use appropriate contraception)

4.2.3 Known allergy to one or more of the study medications

4.2.4 Known contraindication to β-blockers (including hypersensitivity; unstable angina; severe asthma; bronchospasm predisposition; symptomatic hypotension; severe heart failure, moderate to high grade conduction disease, bradycardia <50 beats per minute, sick sinus syndrome, severe cardiomegaly, phaeochromocytoma)

4.2.5 Known contraindication to celecoxib (NSAID associated asthma or urticaria; concomitant NSAIDs (excluding low dose aspirin); active peptic ulcer with GI bleed; eGFR <30mls/min; renal impairment with eGFR<30mL/min

4.2.6 Patient already taking a β blocker, COX2 inhibitor (excluding low dose aspirin; ie ≤150mg daily), or digoxin within one month of study entry

4.2.7 Immune suppression (including transplant recipients, chronic haematological malignancies, immune-suppressive medications for autoimmune disorders)

4.2.8 Internal malignancy (other than metastatic melanoma) within the past 5 years

4.2.9 Current use of non-dihydropyridine calcium channel blockers (verapamil or diltiazem) or amiodarone

4.2.10 Current use of anti-platelets agents (aside from aspirin) i.e. P2Y12 antagonist (clopidogrel, ticagrelor or prasugrel)

4.2.11 Liver failure (cirrhosis)

4.2.12 Treatment with systemic immunotherapy (ipilimumab and/or PD1 inhibitor) within 4 weeks of surgery

# **5. STUDY PROCEDURES**

### **5.1. Participants N = 40 e**ligible patients with nodal and/or in transit metastatic melanoma randomised 1:1 to ProCel regimen or control, with stratification by tumour site (nodal disease ± skin or skin metastases only), and history of systemic immunotherapy or not. Eligibility will also be dependent on a baseline ECG with cardiologist ECG review confirming suitability for, and no ECG contraindications to, oral propranolol.

### **5.2. Investigation Plan**

Eligible participants randomised to ProCel will receive propranolol in combination with celecoxib for five days preoperatively, day of surgery and for 14 days postoperatively:

The study drugs will be sourced through the RPA Investigational Drugs Unit pharmacy.

**Celecoxib:** 200 mg/bd D1-20 [equivalent to etodolac 400bd]; participants will be provided with two bottles of 30 x 200mg tabs

**Propranolol:** participants will be provided with two bottles of 100 x 10mg tablets

D1-D2 Propranolol 20mg bd

D3-D5 Propranolol 40mg bd

D6 ***SURGERY***  Propranolol 60mg bd

(baseline blood pressure, heart rate recorded preoperatively on day of surgery; anaesthetist review preoperatively on day of surgery as per standard clinical care)

D7-D13 (D13=7 days postop)Propranolol 40mg bd

D14-D20 (D20=14d postop) Propranolol 20mg bd

(wound review and tablet adherence assessment at D20)

Participants will be provided with a pulse oximeter to record their heart rate each day. If heart rate is <60 beats per minute, participants are advised to continue the current dose, not increase the dose, and to notify the research team. If participants experience dizziness or shortness of breath, they are advised to stop taking propranolol, and to notify the research team. If the treating anaesthetist determines heart rate and blood pressure are too low for safe anaesthesia, surgery will not proceed.

Excised tumour will be used for routine histopathology, as well as for marker studies using imaging mass cytometry to assess tumour immunology and proliferation. This technique enables assessment of approximately 40 immune, proliferation and angiogenesis and apoptosis markers in each 1mm tissue core. Markers may include, but need not be limited to, markers such as B2M-c, CD11c, CD14, CD141, CD163, CD16a-c, CD1c-c, CD20, CD27-c, CD31, CD34, CD38, CD39, CD3e, CD4, CD45, CD45RO, CD68, CD8, CollagenIV, FOXP3, GZMB, HLA-A, HLA-DR, HLA-E, ICOS, IDO1, Ki-67, LAG3, MITF-c, MLANA-c, PD-1, PD-L1, PMEL-c, S100B-c, SMA, SOX10-c, TBET-c, TCF7-c, TIM3-c, Vimentin and VISTA.

Routine blood samples will be taken 5-28 days preoperatively (before the start of ProCel treatment) for routine testing (full blood count, electrolytes, creatinine, liver function tests and any other bloods that are clinically relevant for individual participants, eg coagulation studies) and also for blood inflammatory and immune markers.

Tumour mutation profiles (BRAF, NRAS) are routinely collected and this information will be examined in control and ProCel arms.

At D15 postoperatively (=D20; range D19-D26), participants will have a blood sample taken for serum inflammatory and immune markers as well as postoperative full blood count, electrolytes, liver function tests and creatinine. Imaging and clinical and histological evidence of recurrence at 4 months postoperatively will be collected as per routine clinical practice.

Participants with metastatic melanoma will be followed up with routine imaging, bloods and clinical examination as per standard clinical practice. Longer term follow-up information looking at recurrence rates in participants will thus continue to be collected in the medium to long term as part of clinical practice.

**Protocol Compliance**

Drug intake and adherence will be monitored by a clinical research coordinator, based on patient reports and counting of unused tablets.

| **Investigation/intervention** | **Timing (Study Day)** | **Notes** |
| --- | --- | --- |
| Informed consent | At/ before D0 | Baseline |
| Inclusion/exclusion criteria | At/before D0 | Baseline |
| Medical History | At/before D0 | Baseline |
| ECG | At/Before D0 | Baseline; for cardiologist review prior to randomisation |
| Concomitant medications | At/before D0 | Baseline; medications checked and any changes noted at each visit |
| Blood sample collected | D0 (or up to 4 wks prior) | Baseline blood sample (total 40mL): for inflammatory and immune markers as per MIA biobank protocol (32mL) plus routine preoperative bloods (FBC, EUC, LFTs*; 8mL). |
| QoL, perceived stress and state optimism scales | D0 |  |
| Nodal and/or skin metastases biopsied (2 x 2mm^2^ core biopsies for diagnosis and for tissue microarray | 28-14 days before D0 |  |
| ProCel treatment | D0 | Commenced 5d preoperatively: (D0) |
| Tumour assessment (IHC) | D6 | Excision specimens obtained after 5d of ProCel regimen and controls (D6 = day of surgery) |
| Blood sample collected | D20 (range D20-D27) | Postoperative bloods collected D20 (range D20-D27; ie 2 to 3 wks postoperatively) for inflammatory and immune markers as per MIA biobank protocol (32mL) and routine postoperative bloods (FBC, EUC, LFTs; 8mL) |
| QoL, perceived stress and state optimism scales | D20 (range D20-D27) |  |
| Adverse events | During and for 30 days after ProCel regimen | D6 (day of surgery), D20 (postop followup), D45 (30 days after ceasing medication) |
| Clinical and radiological followup | 4 months postoperatively (range 3-5 months) | Clinical review and surveillance imaging as per standard clinical practice |
| QoL, perceived stress and state optimism scales | 4 months postoperatively(range 3 – 5 months) | Performed at the time of 4 month postoperative review |

### **FBC, full blood count; EUC, electrolytes urea creatinine; LFTs, liver function tests*

**Quality of Life Questionnaires**

Participants will be asked to complete quality of life questionnaires at baseline, D20 and at 4 months post operatively.

Questionnaires to be used:

- EORTC QLQ-C30^40^
- MCQ-28^41^
- State Optimism Measure^42^
- Perceived Stress Scale^43^

### **5.3. Participant Recruitment and Enrolment**

Participants will be recruited from the Melanoma and Surgical Oncology clinics at RPA Hospital and Poche Centre. Potential participants, who meet all inclusion and no exclusion criteria will be provided with the participant information statement and consent form at their first appointment. Participants will have at least one week until their next appointment to consider whether they wish to participate. Participants who complete the informed consent process appropriately with their treating clinician at their second appointment will be enrolled in the study. Potential participants will have medical and medication history, blood pressure, heart rate and ECG reviewed by a cardiologist prior to randomisation to ensure their suitability for the study.

### **5.4. Randomisation Procedure**

Randomisation will be performed via RedCap, with participants stratified by nodal disease ± skin or skin metastases only; previous systemic immunotherapy versus no previous systemic immunotherapy

### **5.5. End of Study Intervention and Withdrawal Procedure**

**5.5.1 Criteria for Treatment Cessation**

Participants may cease to take part in the study in the following circumstances:

- Intercurrent illness which would, in the judgement of the investigator, affect assessments of clinical status to a significant degree, and require discontinuation of protocol therapy.
- Unacceptable toxicity or reaction.
- Participation in this study is voluntary; participants are able to withdraw at any time. Those who withdraw from the study will be asked to continue to attend follow up visits, as per routine clinical practice, to allow the collection of outcome data and to enable appropriate ongoing clinical management and follow up. If a participant decides to stop their follow up visits, their health status will be periodically ascertained via phone contact with their referring doctor or by direct phone contact with the patient.
- Completion of therapy. Efforts should be made to maintain the investigations schedule and continue follow up, even if patients discontinue protocol treatment prematurely and/or no longer attend the clinic.

# **6. SAFETY**

Adverse events will be recorded at each visit and graded using the NCI Common Terminology Criteria for Adverse Events Version 4.0 (CTCAE).

### **6.1. Serious Adverse Event Reporting**

Any SAEs which occur during participation in the study should be reported to the PI in the manner described below. In addition, the local Health Research Ethics Committee (HREC) should be notified.

### **6.2. Adverse Events**

6.2.1 Any death (i.e. grade 5 event) which occurs while a patient is receiving protocol treatment or occurring within 30 days of stopping study agent must be reported on a Serious Adverse Event Report (SAE) form and sent to the PI within 10 working days of the event. This form must be signed by the responsible investigator.

6.2.2 All grade 4 unexpected reactions occurring within 30 days of stopping study treatment must be reported on an SAE form to the PI within 10 working days of the event. This form must be signed by the responsible investigator.

# **7. CONFIDENTIALITY AND STORAGE AND ARCHIVING OF STUDY**

Participant data will be stored and managed securely, as both hard and soft copy, on-site at RPAH. All data collected will be confidential and will not be accessed by anyone outside those directly involved in the coordination of the study unless necessary for patient safety. Treatment of data will be consistent with patient confidentiality in the management of medical records.

# **8. TRIAL SPONSORSHIP AND FINANCING**

This is an investigator-initiated study. The Sydney Local Health District (SLHD) is the sponsor of RPAH. Additional sites will be sponsored by their own local health district or institution and will take on all sponsor-related liabilities. Surgical care, tissue histopathology and imaging investigations are as per routine clinical care, and the cost of study consumables (propranolol and celecoxib) as well as for tumour marker studies and inflammatory blood marker studies and trial management costs will be met from research funds. There is no involvement nor financial contribution from the drug manufacturers. Statistical support will be provided by MIA.

**9. AUDITS**

The investigators will permit study-related monitoring, audits, HREC review, and regulatory agency inspections, and will provide direct access to source documents.

The schedule of planned GCP audits is outlined below:

| **Task** | **Person responsible** | **Frequency** |
| --- | --- | --- |
| Study staff GCP certificates up to date | Katina Selvaraj | Yearly |
| Consent forms signed | Katina Selvaraj | Monthly |
| Protocol deviations and serious adverse events reported to HREC | Robyn Saw | Quarterly / when required |
| Annual reports submitted to HREC | Robyn Saw | Yearly |
| Communications with HREC and site governance | Katina Selvaraj | Quarterly / when required |
| Management of the investigational medicinal product (IMP) used in the trial | Katina Selvaraj | Weekly |

# **10. DATA SAFETY MONITORING BOARD (DSMB)**

A DSMB will be convened involving:

1. Dr Jenny Lee – Chair of the DSMB. Experienced medical oncologist and researcher at Lifehouse.
2. A/Prof Jonathan Hong – Colorectal surgeon and researcher RPAH, Institute of Academic Surgery and University of Sydney
3. Dr Mark Porter – Anaesthetist RPAH

Data to review:

1. Information on participant screening
2. Total recruitment
3. Eligibility violations
4. Baseline characteristics (demographics, measurements including heart rate, previous health status of participants)
5. Participants withdrawn
6. Compliance with treatment schedule and intervention
7. Completion of scheduled visits
8. Completeness of follow up and data collection
9. (Serious) adverse events reporting
10. Planned or implemented protocol amendments

Meetings:

1. prior to start of patient recruitment
2. at 50% of trial recruitment target (ie when 10 patients have been enrolled – at least 5 in intervention arm)
3. end of recruitment of patients
4. at the request of the investigators or HREC
5. in the event of a serious adverse event

# **11. REFERENCES**

1. Gershenwald JE, Scolyer RA, Hess KR, et al. Melanoma staging: Evidence-based changes in the American Joint Committee on Cancer eighth edition cancer staging manual. CA: a cancer journal for clinicians 2017;67:472-92.

2. Miyazono F, Natsugoe S, Takao S, et al. Surgical maneuvers enhance molecular detection of circulating tumor cells during gastric cancer surgery. Annals of surgery 2001;233:189-94.

3. Weitz J, Herfarth C. Surgical strategies and minimal residual disease detection. Seminars in surgical oncology 2001;20:329-33.

4. Eschwège P, Dumas F, Blanchet P, et al. Haematogenous dissemination of prostatic epithelial cells during radical prostatectomy. Lancet 1995;346:1528-30.

5. Yamaguchi K, Takagi Y, Aoki S, Futamura M, Saji S. Significant detection of circulating cancer cells in the blood by reverse transcriptase-polymerase chain reaction during colorectal cancer resection. Annals of surgery 2000;232:58-65.

6. Abramovitch R, Marikovsky M, Meir G, Neeman M. Stimulation of tumour growth by wound-derived growth factors. Br J Cancer 1999;79:1392-8.

7. Hofer SO, Molema G, Hermens RA, Wanebo HJ, Reichner JS, Hoekstra HJ. The effect of surgical wounding on tumour development. European journal of surgical oncology : the journal of the European Society of Surgical Oncology and the British Association of Surgical Oncology 1999;25:231-43.

8. Svendsen MN, Werther K, Nielsen HJ, Kristjansen PE. VEGF and tumour angiogenesis. Impact of surgery, wound healing, inflammation and blood transfusion. Scandinavian journal of gastroenterology 2002;37:373-9.

9. Curigliano G, Petit JY, Bertolini F, et al. Systemic effects of surgery: quantitative analysis of circulating basic fibroblast growth factor (bFGF), Vascular endothelial growth factor (VEGF) and transforming growth factor beta (TGF-beta) in patients with breast cancer who underwent limited or extended surgery. Breast cancer research and treatment 2005;93:35-40.

10. Lutgendorf SK, Cole S, Costanzo E, et al. Stress-related mediators stimulate vascular endothelial growth factor secretion by two ovarian cancer cell lines. Clin Cancer Res 2003;9:4514-21.

11. Greenfeld K, Avraham R, Benish M, et al. Immune suppression while awaiting surgery and following it: dissociations between plasma cytokine levels, their induced production, and NK cell cytotoxicity. Brain, behavior, and immunity 2007;21:503-13.

12. Bartal I, Melamed R, Greenfeld K, et al. Immune perturbations in patients along the perioperative period: alterations in cell surface markers and leukocyte subtypes before and after surgery. Brain, behavior, and immunity 2010;24:376-86.

13. Sietses C, Beelen RH, Meijer S, Cuesta MA. Immunological consequences of laparoscopic surgery, speculations on the cause and clinical implications. Langenbecks Arch Surg 1999;384:250-8.

14. Hiller JG, Perry NJ, Poulogiannis G, Riedel B, Sloan EK. Perioperative events influence cancer recurrence risk after surgery. Nat Rev Clin Oncol 2018;15:205-18.

15. Wang D, Dubois RN. Eicosanoids and cancer. Nat Rev Cancer 2010;10:181-93.

16. Chiu LC, Tong KF, Ooi VE. Cytostatic and cytotoxic effects of cyclooxygenase inhibitors and their synergy with docosahexaenoic acid on the growth of human skin melanoma A-375 cells. Biomedicine & pharmacotherapy = Biomedecine & pharmacotherapie 2005;59 Suppl 2:S293-7.

17. Tilley SL, Coffman TM, Koller BH. Mixed messages: modulation of inflammation and immune responses by prostaglandins and thromboxanes. J Clin Invest 2001;108:15-23.

18. Buvanendran A, Kroin JS, Berger RA, et al. Upregulation of prostaglandin E2 and interleukins in the central nervous system and peripheral tissue during and after surgery in humans. Anesthesiology 2006;104:403-10.

19. Yang EV, Sood AK, Chen M, et al. Norepinephrine up-regulates the expression of vascular endothelial growth factor, matrix metalloproteinase (MMP)-2, and MMP-9 in nasopharyngeal carcinoma tumor cells. Cancer Res 2006;66:10357-64.

20. Thaker PH, Han LY, Kamat AA, et al. Chronic stress promotes tumor growth and angiogenesis in a mouse model of ovarian carcinoma. Nature medicine 2006;12:939-44.

21. Masur K, Niggemann B, Zanker KS, Entschladen F. Norepinephrine-induced migration of SW 480 colon carcinoma cells is inhibited by beta-blockers. Cancer Res 2001;61:2866-9.

22. Melamed R, Rosenne E, Shakhar K, Schwartz Y, Abudarham N, Ben-Eliyahu S. Marginating pulmonary-NK activity and resistance to experimental tumor metastasis: suppression by surgery and the prophylactic use of a beta-adrenergic antagonist and a prostaglandin synthesis inhibitor. Brain, behavior, and immunity 2005;19:114-26.

23. Fitzgerald PJ. Is norepinephrine an etiological factor in some types of cancer? Int J Cancer 2009;124:257-63.

24. Yang EV, Kim SJ, Donovan EL, et al. Norepinephrine upregulates VEGF, IL-8, and IL-6 expression in human melanoma tumor cell lines: implications for stress-related enhancement of tumor progression. Brain, behavior, and immunity 2009;23:267-75.

25. Fitzgerald PJ. Testing whether drugs that weaken norepinephrine signaling prevent or treat various types of cancer. Clinical epidemiology 2010;2:1-3.

26. De Giorgi V, Grazzini M, Gandini S, et al. Treatment with β-blockers and reduced disease progression in patients with thick melanoma. Archives of internal medicine 2011;171:779-81.

27. Lemeshow S, Sørensen HT, Phillips G, et al. β-Blockers and survival among Danish patients with malignant melanoma: a population-based cohort study. Cancer Epidemiol Biomarkers Prev 2011;20:2273-9.

28. Elmets CA, Viner JL, Pentland AP, et al. Chemoprevention of nonmelanoma skin cancer with celecoxib: a randomized, double-blind, placebo-controlled trial. J Nat Cancer Inst 2010;102:1-10.

29. Khalili JS, Hwu P, Lizée G. Forging a link between oncogenic signaling and immunosuppression in melanoma. Oncoimmunology 2013;2:e22745.

30. Botti G, Fratangelo F, Cerrone M, et al. COX-2 expression positively correlates with PD-L1 expression in human melanoma cells. Journal of translational medicine 2017;15:46.

31. Iacono D, Cinausero M, Gerratana L, et al. Tumour-infiltrating lymphocytes, programmed death ligand 1 and cyclooxygenase-2 expression in skin melanoma of elderly patients: clinicopathological correlations. Melanoma Res 2018;28:547-54.

32. Soares CD, Borges CF, Sena-Filho M, et al. Prognostic significance of cyclooxygenase 2 and phosphorylated Akt1 overexpression in primary nonmetastatic and metastatic cutaneous melanomas. Melanoma Res 2017;27:448-56.

33. Meyer S, Vogt T, Landthaler M, et al. Cyclooxygenase 2 (COX2) and Peroxisome Proliferator-Activated Receptor Gamma (PPARG) Are Stage-Dependent Prognostic Markers of Malignant Melanoma. PPAR research 2009;2009:848645.

34. Panza E, De Cicco P, Ercolano G, et al. Differential expression of cyclooxygenase-2 in metastatic melanoma affects progression free survival. Oncotarget 2016;7:57077-85.

35. Minami S, Lum CA, Kitagawa KM, Namiki TS. Immunohistochemical expression of cyclooxygenage-2 in melanocytic skin lesions. Int J Dermatol 2011;50:24-9.

36. Subbaramaiah K, Dannenberg AJ. Cyclooxygenase 2: a molecular target for cancer prevention and treatment. Trends Pharmacol Sci 2003;24:96-102.

37. Gilron I, Milne B, Hong M. Cyclooxygenase-2 inhibitors in postoperative pain management: current evidence and future directions. Anesthesiology 2003;99:1198-208.

38. Tudor DV, Bâldea I, Lupu M, et al. COX-2 as a potential biomarker and therapeutic target in melanoma. Cancer biology & medicine 2020;17:20-31.

39. Haldar R, Ricon-Becker I, Radin A, et al. Perioperative COX2 and β-adrenergic blockade improves biomarkers of tumor metastasis, immunity, and inflammation in colorectal cancer: A randomized controlled trial. Cancer 2020;126:3991-4001.

40. Aaronson NK, Ahmedzai S, Bergman B, Bullinger M, Cull A, Duez NJ, Filiberti A, Flechtner H, Fleishman SB, de Haes JC, et al. The European Organization for Research and Treatment of Cancer QLQ-C30: a quality-of-life instrument for use in international clinical trials in oncology. J Natl Cancer Inst 1993; 85:365-76.

41. Winstanley J, White E, Saw R, et al. Development of the Melanoma Concerns

Questionnaire(c) (MCQ-28(c)); refinement of the EORTC QLQ-MEL38 Module. Psychooncology 2020 Feb;29(2):321-330. doi: 10.1002/pon.5251. Epub 2019 Dec 4.

42. Millstein, R. A., Chung, W. J., Hoeppner, B. B., Boehm, J. K., Legler, S. R., Mastromauro, C. A., & Huffman, J. C. Development of the State Optimism Measure. Gen Hosp Psychiatry, 2019;58, 83-93. doi:10.1016/j.genhosppsych.2019.04.002.

43. Cohen, S., Kamarck, T., & Mermelstein, R. A Global Measure of Perceived Stress. Journal of Health and Social Behavior, 1983;24(4), 385-396. doi:10.2307/2136404.

**Appendix 1. Quality of Life Questionnaires**

**European Organisation for Research and Treatment of Cancer Quality of Life Questionnaire Version 3 (EORTC QLQ-C30)**

We are interested in some things about you and your health. Please answer all of the questions yourself by clicking on the best response. There are no "right" or "wrong" answers.

|  |  | **Not at all** | **A little bit** | **Quite a bit** | **Very much** |
| --- | --- | --- | --- | --- | --- |
| 1. | Do you have any trouble doing strenuous activities, like carrying a heavy shopping bag or a suitcase? | 1 | 2 | 3 | 4 |
| 2. | Do you have any trouble taking a long walk? | 1 | 2 | 3 | 4 |
| 3. | Do you have any trouble taking a short walk outside of the house | 1 | 2 | 3 | 4 |
| 4. | Do you need to stay in bed or a chair during the day? | 1 | 2 | 3 | 4 |
| 5. | Do you need help with eating, dressing, washing yourself or using the toilet? | 1 | 2 | 3 | 4 |

**During the past week:**

|  |  | **Not at all** | **A little bit** | **Quite a bit** | **Very much** |
| --- | --- | --- | --- | --- | --- |
| 6. | Were you limited in doing either your work or other daily activities? | 1 | 2 | 3 | 4 |
| 7. | Were you limited in pursuing your hobbies or other leisure time activities? | 1 | 2 | 3 | 4 |
| 8. | Were you short of breath? | 1 | 2 | 3 | 4 |
| 9. | Have you had pain? | 1 | 2 | 3 | 4 |
| 10. | Did you need to rest? | 1 | 2 | 3 | 4 |
| 11. | Have you had trouble sleeping? | 1 | 2 | 3 | 4 |
| 12. | Have you felt weak? | 1 | 2 | 3 | 4 |
| 13. | Have you lacked appetite? | 1 | 2 | 3 | 4 |
| 14. | Have you felt nauseated? | 1 | 2 | 3 | 4 |
| 15. | Have you vomited? | 1 | 2 | 3 | 4 |
| 16. | Have you been constipated? | 1 | 2 | 3 | 4 |
| 17. | Have you had diarrhea? | 1 | 2 | 3 | 4 |
| 18. | Were you tired? | 1 | 2 | 3 | 4 |
| 19. | Did pain interfere with your daily activities? | 1 | 2 | 3 | 4 |
| 20. | Have you had difficulty in concentrating on things, like reading a newspaper or watching television? | 1 | 2 | 3 | 4 |
| 21. | Did you feel tense? | 1 | 2 | 3 | 4 |
| 22. | Did you worry? | 1 | 2 | 3 | 4 |
| 23. | Did you feel irritable? | 1 | 2 | 3 | 4 |
| 24. | Did you feel depressed? | 1 | 2 | 3 | 4 |
| 25. | Have you had difficulty remembering things? | 1 | 2 | 3 | 4 |
| 26. | Has your physical condition or medical treatment interfered with your family life? | 1 | 2 | 3 | 4 |
| 27. | Has your physical condition or medical treatment interfered with your social activities? | 1 | 2 | 3 | 4 |
| 28. | Has your physical condition or medical treatment caused you financial difficulties? | 1 | 2 | 3 | 4 |

**For the following questions please circle the number between 1 and 7 that best applies to you**

| 29. | How would you rate your overall health during the past week? | | | | | | |
| --- | --- | --- | --- | --- | --- | --- | --- |
|  | 1 | 2 | 3 | 4 | 5 | 6 | 7 |
|  | Very poor |  |  |  |  |  | Excellent |
| 30. | How would you rate your overall quality of life during the past week? | | | | | | |
|  | 1 | 2 | 3 | 4 | 5 | 6 | 7 |
|  | Very poor |  |  |  |  |  | Excellent |

*End of EORTC QLQ-C30*

**Melanoma Concerns Questionnaire (MCQ-28)**

Patients sometimes report that they have the following concerns after their diagnosis and treatment for melanoma. Please indicate the extent to which you have experienced these symptoms or concerns.

Please answer by selecting the option that best applies to you.

| **Have you had surgery for your melanoma in the last 12 months?**   - Yes – please continue - No – go to question 6 | | **Not at all** | **A little bit** | **Quite a bit** | **Very much** |
| --- | --- | --- | --- | --- | --- |
| 1. | \| Have you had swelling near your melanoma site? \| \| --- \| | 1 | 2 | 3 | 4 |
| 2. | \| Have you had numbness at the site of your melanoma? \| \| --- \| | 1 | 2 | 3 | 4 |
| 3. | \| Have you had problems with pain at or near your melanoma site? \| \| --- \| | 1 | 2 | 3 | 4 |
| 4. | \| How much have you worried about complications due to your melanoma surgery? \| \| --- \| | 1 | 2 | 3 | 4 |
| 5. | \| How much have you worried about the length of time needed for your melanoma surgery to heal? \| \| --- \| | 1 | 2 | 3 | 4 |

| **Since diagnosis and treatment of your melanoma** | | **Not at all** | **A little bit** | **Quite a bit** | **Very much** |
| --- | --- | --- | --- | --- | --- |
| 6. | \| Have you worried about the increased risk of melanoma for other members of your family? \| \| --- \| | 1 | 2 | 3 | 4 |
| 7. | \| Have you had any regrets about your exposure to sunshine in the past? \| \| --- \| | 1 | 2 | 3 | 4 |
| 8. | \| Have you felt concerned about conducting outdoor activities (e.g. working, swimming, walking, sport) which may result in exposure to the sun? \| \| --- \| | 1 | 2 | 3 | 4 |
| 9. | \| Have you considered making any significant changes to how you will live your life in the future? \| \| --- \| | 1 | 2 | 3 | 4 |
| 10. | \| Have you worried whilst waiting for results of medical tests? \| \| --- \| | 1 | 2 | 3 | 4 |
| 11. | Have you felt hopeful for the future? | 1 | 2 | 3 | 4 |
| 12. | Have you felt able to face the challenges ahead? | 1 | 2 | 3 | 4 |
| 13. | Have you felt able to cope with your diagnosis of melanoma? | 1 | 2 | 3 | 4 |
| 14. | Have you felt able to deal with the shock of being diagnosed with melanoma? | 1 | 2 | 3 | 4 |
| 15. | Have you felt able to carry on with things as normal? | 1 | 2 | 3 | 4 |
| 16. | Have you felt able to feel positive? | 1 | 2 | 3 | 4 |

| **During the past 4 weeks** | | **Not at all** | **A little bit** | **Quite a bit** | **Very much** |
| --- | --- | --- | --- | --- | --- |
| 17. | How much has your melanoma doctor supported you? | 1 | 2 | 3 | 4 |
| 18. | How much have your other healthcare professionals (e.g. nurse) supported you? | 1 | 2 | 3 | 4 |
| 19. | How much have your family members supported you? | 1 | 2 | 3 | 4 |
| 20. | How much has your primary care doctor supported you? | 1 | 2 | 3 | 4 |
| 21. | Have you felt able to contact the melanoma clinical staff if you needed to? | 1 | 2 | 3 | 4 |
| 22. | Have you felt confident that a psychological support service would be available if you needed it? | 1 | 2 | 3 | 4 |
| 23. | Have you been given enough time to think about the treatment options available to you? | 1 | 2 | 3 | 4 |
| 24. | Have you received realistic and reliable information about the extent (spread) of your disease? | 1 | 2 | 3 | 4 |
| 25. | Have you felt confused by information about your diagnosis or treatment from different sources (e.g. internet)? | 1 | 2 | 3 | 4 |
| 26. | Have you had problems in understanding information given about your likely survival? | 1 | 2 | 3 | 4 |
| 27. | How much have those important to you been included in discussions about your treatment options? | 1 | 2 | 3 | 4 |
| 28. | Have you felt confident that your healthcare team communicate with you in a professional manner? | 1 | 2 | 3 | 4 |

*End of MCQ-28*

**State Optimism Measure ^42^**

***Please answer the following items based on how you feel right now, that is, at the present moment using the scale below. Try to answer each item as accurately as possible based on your response to that item alone, without regard to your answers to any previous items.***

***__________________________________________________________________***

***1 2 3 4 5***

***Strongly disagree Strongly agree***

1. ***I am feeling optimistic about life challenges.***
2. ***Right now, I expect things to work out for the best.***
3. ***I am feeling optimistic about my future.***
4. ***I feel that something good will happen today (in the next 24 hours)***
5. ***The future is looking bright to me.***
6. ***At the moment, I expect more to go right than wrong when it comes to my future.***
7. ***I am expecting things to turn out well.***

**Perceived Stress Scale^41^**

The questions in this scale ask you about your feelings and thoughts during the last month. In each case, you will be asked to indicate how often you felt or thought a certain way.

In the last month, how often have you...

|  | Never | Almost never | Sometimes | Fairly often | Very often |
| --- | --- | --- | --- | --- | --- |
| **Been upset because of something that happened unexpectedly?** | **0** | **1** | **2** | **3** | **4** |
| **Felt that you were unable to control the important things in your life?** | **0** | **1** | **2** | **3** | **4** |
| **Felt nervous and “stressed”?** | **0** | **1** | **2** | **3** | **4** |
| **Felt confident about your ability to handle your personal problems?** | **4** | **3** | **2** | **1** | **0** |
| **Felt that things were going your way?** | **4** | **3** | **2** | **1** | **0** |
| **Found that you could not cope with all the things that you had to do?** | **0** | **1** | **2** | **3** | **4** |
| **Been able to control irritations in your life?** | **4** | **3** | **2** | **1** | **0** |
| **Felt that you were on top of your things?** | **4** | **3** | **2** | **1** | **0** |
| **Been angered because of things that were outside of your control?** | **0** | **1** | **2** | **3** | **4** |
| **Felt difficulties were piling up so high that you could not overcome them?** | **0** | **1** | **2** | **3** | **4** |

**Appendix 2. Laboratory tests for Procel**

Blood samples

1. Routine blood samples will be taken 5-28 days preoperatively (before the start of any ProCel treatment) by Douglas Hanly Moir Pathology or RPA for routine testing (full blood count, electrolytes, creatinine, liver function tests and any other bloods that are clinically relevant for individual participants, eg coagulation studies; total 8mL).

2. Inflammatory and immune activation evaluation by cytometry. An additional 32mL of blood will be collected during the same venesections (as per Melanoma Institute Australia biobank protocol) preoperatively and 2 week postoperative bloods by Douglas Hanly Moir/ RPA pathology. Blood collected for cytometry is forwarded to the Melanoma Institute Australia Biobank for storage prior to analysis.

Biopsy and excised tissue samples

1. Routine histopathology (formalin-fixed paraffin embedded (FFPE) tissue) on preoperative metastatic nodes and/or cutaneous in transit metastases (2 x 2mm^2^ core biopsies)
2. Tumour mutation profiles (BRAF, NRAS) – immunohistochemistry (IHC) and formal testing (may have been performed previously – no need to repeat)
3. Two x 1mm^2^ samples will be taken from preoperative core biopsy FFPE tumour blocks for each participant. High dimensional tissue imaging to assess immunological response using an immune-oncology panel will be performed for each sample.
4. Two x 1mm^2^ samples will be taken from the FFPE tissue blocks of nodal and in transit metastatic disease removed at surgery, with tissue microarray creation. Operative samples will be compared to the preoperative (pre-ProCel) tissue samples.
